# In Vivo Vortex Imaging of Bladder

**DOI:** 10.31662/jmaj.2023-0134

**Published:** 2023-11-16

**Authors:** Hideki Mizuno, Seiji Matsumoto, Tokunori Yamamoto

**Affiliations:** 1 Department of Urology, Japanese Red Cross Aichi Medical Center Nagoya Daini Hospital, Nagoya, Japan; 2 Clinical Research Support Center, Asahikawa Medical University Hospital, Asahikawa, Japan; 3 Promotion Office for Open Innovation, Institutes of Innovation for Future Society, Nagoya University, Nagoya, Japan

**Keywords:** urine flow, vortex, wireless capsule endoscope, device, rabbit

The recent development of imaging methods has made it possible to observe and visualize the changes in the shape of the bladder and analyze the fluid dynamics during urination ^(1), (2), (3)^. Turbulence and vortices are presumed to be generated in the bladder because of complex shape changes and rapid urine flow, leading to changes in intravesical pressures during voiding. However, there is no report on in vivo visualization of urine flow. Herein, we performed imaging of intravesical vortices using wireless capsule endoscopes (WCEs).

Experimental evaluation of WCEs in the bladder during urination was performed in female rabbits. Six mature female rabbits (Tokyo Laboratory Animal Science, Tokyo, Japan) weighing 3-4 kg were used during the experimental period. The rabbits were handled in accordance with the Principles of Laboratory Animal Care of the National Institutes of Health. The experimental protocol was approved by the Animal Use Committee of Nagoya University School of Medicine (No. 30,314). Surgical procedures were performed on the rabbits under sodium isoflurane anesthesia under sterile conditions. WCE ^(4)^ and a catheter were inserted after the incision of the bladder dome. Images were continuously transmitted at 4 frames per second to a laptop computer and processed using proprietary software. Saline was pumped into the bladder. We used air (injection by syringe) as the urine flow tracer. The WCE, PillCam™ SB2 (Covidien Japan, Inc., Tokyo, Japan), which has received regulatory approval from the Pharmaceuticals and Medical Devices Agency in Japan, is a medical device that performs imaging of the small intestinal mucosa and provides images for diagnosing diseases of the small intestine. This second-generation device, which weighs <4 g and measures 11 × 26 mm, has high resolution and variable frame rates of up to 6 frames per second while going through fast areas such as the duodenum and down to 2 frames per second when stationary or moving slowly. It has a broader angle of view, 156°, an effective visibility distance of 30 mm, and better optics with automatic light control.

The WCE was efficiently deployed and manually manipulated within the bladder. Complete real-time bladder mucosa image transmission was captured and visualized. The urine flow rotated clockwise from ventral to dorsal at the bladder neck (BN) during urination, and air bubble showed a vortex with clockwise rotation in a crescent moon shape. Initially, air bubble was visualized at the closed BN before urination, and then they moved into a vortex with crescent-shaped clockwise rotation when the BN began to open (Figure 1A, B, C, D, E). The epithelial cluster (collapsed mucosal bladder tissue) was visualized before urination and smoothed out during urination (Figure 2A, B, C). A similar phenomenon was observed in all rabbits; however, differences were observed in the size of each vortex.

**Figure 1.**
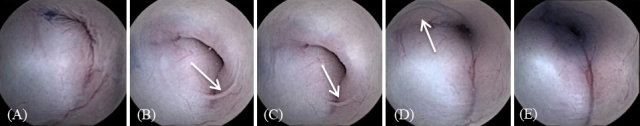
Dynamic imaging of urine flow at the bladder neck by the air bubble (A, B, C, D, E). (A) The closed bladder neck before urination; (B) air bubble rotated from 4 o’clock position (C) to 6 o’clock (D) and 11 o’clock position and (E) sucked into the internal urethra until it disappeared.

**Figure 2.**
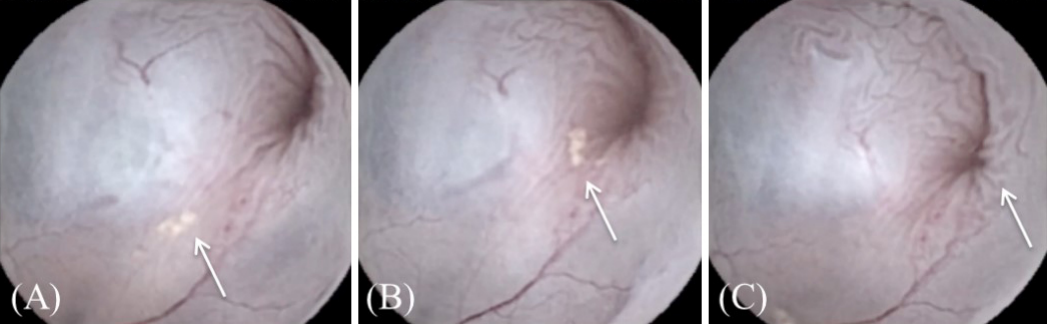
Dynamic imaging of urine flow at the bladder neck by the epithelial cluster (A, B, C). (A) Appearance of the epithelial cluster (B, C) and its gradual disappearance during voiding.

With the recent development of medical equipment and advances in information and communication technology, attempts have been made to acquire biological information using various methods. Several researchers have reported the success of imaging studies to observe and visualize the changes in the shape of the bladder and to analyze the fluid dynamics during urination ^(1), (2), (3)^. We previously used a sensor telemetry system placed in the bladders of rabbits ^(5)^. As a result, we reported that intravesical pressure and heart rate could be monitored continuously in real time. Herein, we aimed to visualize the fluid dynamics of urination in the rabbit bladder. Therefore, we report the first visualization of a vortex at the rabbit BN. Using this device, urine flow could be visualized as a spatiotemporal asymmetric vortex at the BN during urination. Soh^2^ visualized a computational fluid dynamic simulation of male urination with real-time images using magnetic resonance imaging, which is a large vortex within the bladder body. Considering this, along with the results of this study, a large longitudinal vortex is generated in the bladder body at the same time as the start of micturition (the BN begins to open), and a horizontal vortex is generated that rotates clockwise in a crescent shape toward the BN in response to the increase in voiding pressures.

In conclusion, urine flow could be visualized as a vortex at the BN during urination, similar to the vortex seen in a washstand. This vortex phenomenon has been proven to have the basic function of expelling urine from the bladder, and cleansing the bladder.

## Article Information

### Conflicts of Interest

None

### Acknowledgement

We would like to thank Dr. Masanao Nakamura and Prof. Hiroki Kawashima (Department of Gastroenterology and Hepatology, Nagoya University Graduate School of Medicine, Nagoya, Japan) for providing us with and teaching us how to use this device as part of their cooperation with this study.

### Author Contributions

Hideki Mizuno contributed to the data acquisition, revised the manuscript, and approved the final version. Seiji Matsumoto contributed to the design and analysis, drafted and revised the manuscript, and approved the final version. Tokunori Yamamoto contributed to the concept, design, and analysis, revised the manuscript, and approved the final version.
